# Feasibility, classification and potential clinical impact of non-invasive delineation of abdominal lymphatic vessels in patients following TCPC with T2 weighted MRI

**DOI:** 10.1038/s41598-024-81299-w

**Published:** 2024-11-29

**Authors:** Anja Hanser, Michael Hofbeck, Melanie Hofmeister, Petros Martirosian, Andreas Hornung, Michael Esser, Fritz Schick, Thomas Küstner, Renate Kaulitz, Jörg Michel, Konstantin Nikolaou, Jürgen Schäfer, Christian Schlensak, Winfried Baden, Johannes Nordmeyer, Ludger Sieverding

**Affiliations:** 1 Department of Pediatric Cardiology, University Children’s Hospital, University of Tübingen, Tübingen, Germany; 2 Section on Experimental Radiology, University Hospital of Tübingen, Tübingen, Germany; 3 Department of Diagnostic and Interventional Radiology, University Hospital of Tübingen, Tübingen, Germany; 4 Medical Image and Data Analysis (MIDAS.lab), Department of Diagnostic and Interventional Radiology, University Hospital of Tübingen, Tübingen, Germany; 5 Department of Cardiothoracic and Vascular Surgery, University Hospital of Tübingen, Tübingen, Germany; 6 Department of Pediatric Cardiology, University Hospital Tübingen, Hoppe-Seyler-Str. 1, 72076 Tübingen, Germany

**Keywords:** TCPC, Fontan Circulation, Abdominal lymphatic vessels, T2-weighted imaging, Diagnosis, Paediatrics, Cardiology

## Abstract

Recent research in patients with functionally univentricular hearts (UVH) is focusing on pathologies of the lymphatic vessels. Morphology of the abdominal lymphatic vessels was analyzed by MRI in patients with UVH following total cavopulmonary connection (TCPC) and it was examined, if clinical and laboratory parameters correlate with changes after TCPC. We prospectively examined 33 patients at the age of 19.8 (14.6;30.2) years [median (Q1;Q3)] after TCPC (follow-up 14.3 years (9.7;24.9) with a heavily T2-weighted MRI sequence on a 3.0 T scanner. Examinations in coronal orientation were performed with respiratory gating, slice thickness 0.6 mm, TR 2400 ms, TE 692 ms, FoV 460 mm (covering thoracic and abdominal regions), scan time 14:41 min (13:18;16:30) after a solid meal and a cup of pineapple juice. The findings were classified according to delineation of abdominal lymphatic vessels. Type 1: <3 abdominal vessels (av) definable; type 2: 4–6 av definable; type 3: >6 av and/or oedematous changes or ascites. The results were correlated with parameters obtained at the annual routine check-up. Statistical analysis was performed using U-test and Chi-square test. Fifteen patients (group 1) showed type 3 lymphatic morphologies, two of which had ascites. Eighteen patients (group 2) showed lower grade morphologies (type 1–2). Image quality was rated considering the delineation of the common hepatic duct and did not differ between groups (*p* = 0.134). “Lymphatic burden” was automatically examined and was indexed to the number of delineated abdominal vessels and showed quantification according to the chosen categories type 1–3. Patients in group 1 were younger at MRI examination (17.4;14.3/18.9 vs. 26.2;18.2/32.3 years, *p* = 0.03). Superior cavopulmonary connection (SCPC) had been performed earlier in group 1 (9.9;7.9/25.5 vs. 29.2;13.7/66.6 months, *p* = 0.018). Laboratory examinations in group 1 showed lower levels for Immunoglobulin G (IgG), Lipase, α-Antitrypsin, Cystatin C and TSH. There were no significant differences for total protein, NTproBNP, lymphocytes or platelets. A history of chylothorax was present in 7/15 versus 2/18 *p* = 0.022. Protein-losing enteropathy (PLE) occurred in 4/15 versus 1/18 (*p* = 0.092). T2 weighted MRI is feasible for noninvasive delineation of abdominal lymphatic vessel in patients following TCPC. In the long-term follow-up, patients with more pronounced changes of the abdominal lymphatic vessels were younger at SCPC and were more likely to show a history of chylothorax and lower IgG values.

## Introduction

**Fig. 1:**
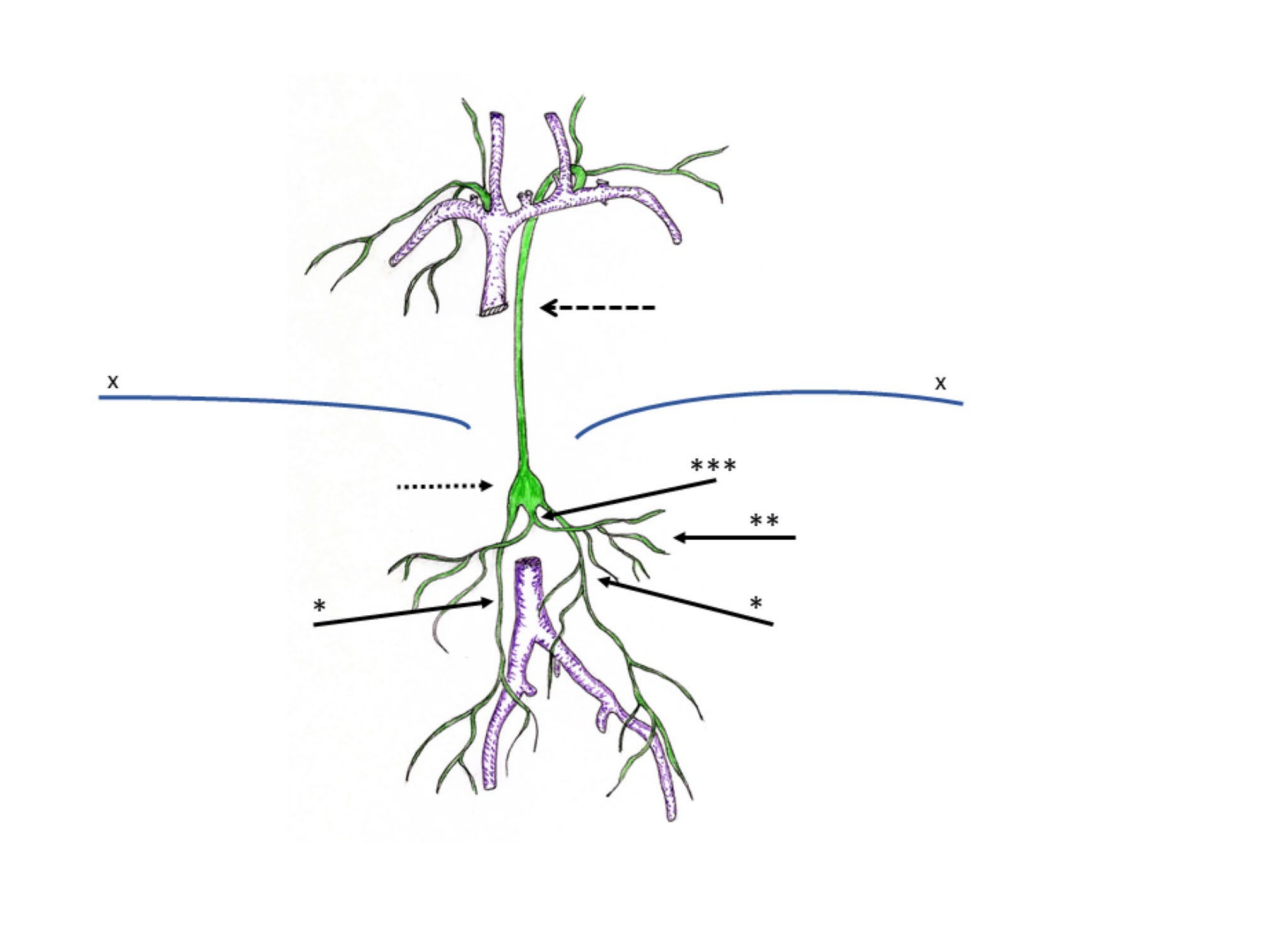
Lymphatic anatomy: The lymph from the mesentery (arrow two stars) is drained into the common intestinal trunk (arrow three stars). The common intestinal trunk is connected with bilateral lumbar trunks (arrow one star) to the cisterna chyli (dotted arrow) from where the thoracic duct (line-dotted arrow) drains to the venous angle. X: diaphragm.

Based on significant improvements in diagnosis and treatment patients with functionally univentricular hearts (UVH) represent a rapidly increasing population with the majority of these patients surviving into adulthood^1–3^. Due to the absence of a second ventricle perfusion of the lungs is achieved by anastomoses of the superior and inferior vena cava with the pulmonary arteries establishing a so-called Fontan circulation (total cavopulmonary connection, TCPC)^1,4,5^. In the absence of a subpulmonary ventricle the driving force of the pulmonary perfusion is based to a large extent on the central venous pressure (CVP), which is always elevated in these patients. During the recent years it became obvious that elevated CVP in patients with Fontan circulation has also major impact on the lymphatic system, resulting in impairment of the lymphatic system both in the thorax and in the abdomen (Fig. 1)^6–12^. Morbidities resulting from alterations of the lymphatic system (chylothorax, chylopericardium, plastic bronchitis and protein losing enteropathy (PLE) have been summarized under the term “Failing Fontan” circulation and have significant impact on life quality and life expectancy^13^. Better understanding of lymphatic abnormalities and their pathophysiology requires imaging modalities that can be applied invasively and non-invasively in clinical practice^14^. Heavily T2 weighted 3D Fast-Spin-Echo (FSE) sequences have been introduced recently as a non-invasive technique for visualization of the lymphatic system^15–18^. In the underlying study, we aimed at noninvasive visualization of abdominal lymphatic vessels in patients with UVH after TCPC and correlated the morphological findings with clinical and laboratory parameters^19,20^. We hypothesized that changes in the abdominal lymphatic system are associated with changes in clinical and laboratory parameters.

## Materials and methods

This prospective study was approved by the ethics committee of Tuebingen University Hospital (project number 873/2017BO1). Written informed consent was obtained from the patients or their legal representatives. All methods were performed in accordance with the relevant guidelines and regulations.

### Study cohort

We performed a prospective study including thirty-three consecutive patients with UVH [median age 19.8 (14.6;30.2) years [Mdn (Q1;Q3)]] following establishment of TCPC [median follow-up 14.3 years (9.7;24.9)] who underwent cardiac MRI between August 20, 2018, and July 5, 2021. In our institution cardiac MRI is part of routine follow-up in patients with UVH following TCPC at least every 5 years. All patients who qualified for MRI during the study period were offered an MRL (magnetic resonance lymphangiography). Exclusion criteria were implanted pacemakers and absent consent of patients and/or legal representatives. The results of the MRI examination were correlated with other data from the annual check-up, including medical history, lymphatic complications as prolonged pleural effusion, PLE and plastic bronchitis, physical examination, echocardiography, sonography of the abdomen, laboratory tests and hemodynamic data derived from cardiac catherization. Exercise tests were performed on the treadmill according to the protocol of the German Society of Pediatric Cardiology^21^. This treadmill protocol includes an increase in speed of 0.5 km/h and an increase in gradient of 3% to a maximum of 21% every 1.5 min. Each increase is termed step. VO_2_ was recorded continuously during exercise, the peak value achieved was taken as VO_2_max and referred to body weight, as VO_2_max/kg (maximal oxygen consumption). O_2_pulse was calculated as maximum oxygen uptake divided by the actual heart rate at the same time (O_2_/HR).

### MR-examination

Subjects were examined in supine position on a 3.0T MRI scanner (MAGNETOM Prisma^Fit^, Siemens Healthcare, Erlangen, Germany). The scan protocol included a routine cardiac MRI examination and in addition a coronal Half-Fourier Acquisition with Single Shot Turbo Spin Echo (HASTE) sequence with breath-holding for chest and abdominal regions. We performed no further abdominal scans. 4D Flow MRI was performed for non-invasive measurement of blood flow. For MR lymphangiography a heavily T2-weighted 3D FSE sequence was applied using respiratory navigator gating with following parameters: isotropic voxel size 1.2 × 1.2 × 1.2 mm^3^, interpolated to 0.6 × 0.6 × 0.6 mm^3^, 224 slices per slab, slab thickness 27 cm, repetition time 2400 ms, echo time 692 ms, field of view 460 × 460 mm, matrix size 384 × 384. Imaging of the neck, chest and abdomen was performed in all patients. All patients were asked to eat a high-fat breakfast and were offered 200 ml of cream (fat content 60 g) 3 hours before the MRI-scan to improve image quality by stimulation of the lymphatic flow^18^. In order to reduce undesired signals from the stomach, the volunteers drank 200 ml of pineapple juice prior to the MR examination as pineapple juice shortens the T2 relaxation time of the gastrointestinal tract^22^. 4D-Flow sequence was performed aminations in coronal orientation with respiratory gating, voxel size 2.5 mm^3^, 64 slices per slab, FoV 300 × 400 × 160 mm^3^ (covering the heart and the large vessels near the heart).

### Image analysis

**Fig. 2:**
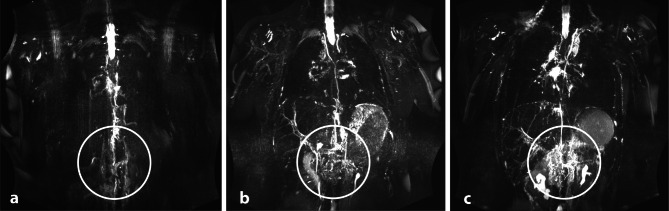
Classification according to the number of delineated abdominal lymphatic vessels (n_av_). (**a**) Type 1: n_av_ < 3 abdominal vessels defineable; (**b**) type 2: n_av_ = 4–6 abdominal vessels defineable; (**c**) type 3 n_av_ > 6 abdominal vessels or oedematous change/ pronounced ascites obscuring the lymphatic vessels.

MR lymphangiograms were reconstructed using targeted maximum-intensity projection (MIP) with a slab thickness of 30 mm. The lymphangiograms were evaluated in accordance by one experienced radiologist (M.E. with 6 years of experience in MRI) and two experienced pediatric cardiologists (A.H.^1^, A.H.^2^, with 5 and 11 years of experience in cardiac MRI), all with special expertise in cardiovascular MRI in CHD. The examiners were blinded for clinical data of the patients, read all MRI studies together. Final decision on classification of the individual patient was obtained by consensus between the three examiners^18^. The abdominal findings were classified according to the number of delineated abdominal lymphatic vessels (n_av_). The individual vessels were counted from the center, near the cisterna chyli. Type 1: n_av_ <3 identifiable; type 2: n_av_ = 4–6 abdominal vessels identifiable; type 3: n_av_ >6 or oedematous changes/ pronounced ascites, obscuring the lymphatic vessels (Fig. 2). Lymphatic abnormalities in the neck and thorax were classified according to Biko et al.^19^. In addition, the vessel area region of interest (ROI) was automatically examined. Signal intensities exceeding 5 standard deviations in the ROI were thresholded to include hyperintense areas with dilated lymphatics^12^. A reader manually corrected the contours by comparing them with the source images, ensuring all visible thoracic lymphatics were included and excluding hyperintense non-lymphatic structures. This manually corrected mask in one plane was similar to the region marked by a circle in Fig. 2. This process provides a surrogate measurement of lymphatic volume, which is then indexed to the number of delineated abdominal lymphatic vessels to quantify the ‘lymphatic burden’^12^.

Results of classification were correlated with parameters obtained at the annual routine check-up. The cross-sectional area of the right and left pulmonary artery were determined in 4D flow measurement. For segmentation of the vessel and determination of the vessel area the postprocessing software GTFlow, Gyrotools^®^ was used.

### Statistical analysis

Statistical analysis was conducted using SPSS, version 28.0 (IBM Corp., Armonk, NY, USA). Distributions of demographic characteristics, cardiac MRI parameters, echocardiographic parameters and laboratory parameters were assessed and expressed by median (Q1;Q3). Differences between patient groups were evaluated by two-sided U-test. A two-sided Chi-square test was used for comparison of clinical variables. A p-value of less than 0.05 indicated a statistically significant difference.

## Results

### Patients demographics, MRI scan and clinical history

From August 2018 until July 2021, a total of 39 MRI examinations were performed in patients after TCPC at our tertiary referral center for children and adults with congenital heart disease. Six patients had to be excluded: Four patients aborted the MRI examination because of the long duration, two examinations were excluded because of excessive artefacts. Thirty patients took a high-fat meal followed by an additional intake of 200 ml cream (fat content 60 g) in twenty-two of them. The others refused the high-fat meal and the intake of cream. Twenty-one patients tolerated the additional cream while one patient reacted with vomiting. The T2-weighted FSE sequence for lymphography was performed without interference in all patients with an acquisition time of 14:41 min (13:18;16:30).

**Fig. 3:**
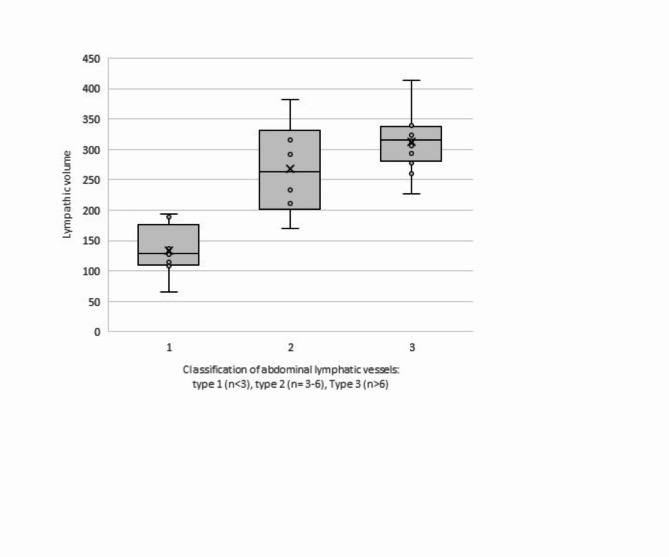
Automatically analysed lymphatic burden indexed to abdominal vessel type 1, type 2, type 3. Vessel area region of interest (ROI) was automatically examined to provide a surrogate measurement of lymphatic volume. Lymphatic burden was indexed to the number of delineated abdominal lymphatic vessels to quantify the lymphatic burden, categorized in abdominal vessel type 1, type 2, type 3. Group 1 includes type 3, group 2 includes type 1 + 2 (in group 1 *n* = 12/15 could be included, in group 2 *n* = 14/18 patients could be included).

Based on the morphology of the abdominal lymphatic system patients were divided into two groups: Group 1 included 15 patients with type 3 lymphatic abnormalities, of which two patients had ascites. Group 2 encompassed 18 patients with less pronounced lymphatic morphologies (type 1–2). Demographic data of both groups are shown in Table 1. Image quality was assessed by the delineation of the common hepatic duct and did not differ between groups (*p* = 0.134). In addition, the ´lymphatic burden` was automatically examined and was indexed to the number of delineated abdominal vessels and showed quantification according to the chosen categories type 1–3 (Fig. 3).

**Table 1:** Demographic variables.

	Group 1 (*n* = 15) median (Q1;Q3)	Group 2 (*n* = 18) median (Q1;Q3)	
Weight (kg)	62.5 (57.8;78.8)	59(51.0;63.0)	*p* = 0.107
Size (m)	1.63(1.58;1.75)	1.71(1.59;1.78)	*p* = 0.310
Sex (f/m)	(6/9)	(6/12)	*p* = 0.696
Age MRI (y)	17.4(14.3;18.9)	26.2(18.2;32.3)	p = 0.030*
Follow-up time (y) (TCPC–MRI)	12.9(9.6;16.1)	23.2(9.7;26.2)	*p* = 0.083
Age SCPC (m)	9.9(8.0;25.6)	29.0(13.7;66.6)	p = 0.018*
Age TCPC (y) one-stage Fontan	3.8(3.2;4.7) *n* = 4	4.2(2.5;6.7) *n* = 7	*p* = 0.691
SPCP-TCPC (m)	30.6(0;41.9)	12.2(0;25.4)	*p* = 0.131
Systemic ventricle:			
RV /LV	5/10	7/11	*p* = 0.741 Chi^2^(1) 0.109

Significant values are with star.

*SCPC* superior cavopulmonary connection, *TCPC* total cavopulmonary connection. *RV* systemic right ventricle, *LV* systemic left ventricle.

In group 1 (*n* = 15) eleven patients had an extracardiac conduit and four patients an intraatrial-lateral tunnel. In group 2 (*n* = 18) seven patients had an extracardiac conduit, nine patients an intraatrial-lateral tunnel and two patients an atriopulmonary anastomosis. Two patients in each group had an initial fenestration in their extracardiac conduit. The systemic ventricle was a left ventricle in 10 of 15 patients (66.7%) in group 1 while 11 of 18 patients (61.1%) had a left ventricle in group 2, *p* = 0.741. None of the patients had required extracorporeal support postoperatively. The median number of cardiac catheter interventions such as stenting of the pulmonary arteries or occlusion of collaterals was 1.0 (0;2) in group 1 versus 0 (0;2) in group 2, *p* = 0.423. Data from cardiac catherizations within a median temporal distance of 2.5 years to the MRI examination were available in 28 patients. They revealed no difference in mean pulmonary artery pressures between both groups [PAPm 11.0 mmHg (9.5;13.5) in group 1 (*n* = 13) versus 12.0 mmHg (8;13) in group 2 (*n* = 15), *p* = 0.853].

Three out of 15 (20%) patients in group 1 received pulmonary vasodilative treatment with sildenafil as compared to 2 out of 18 (11%) patients in group 2, *p* = 0.478. 7 out of 15 (47%) patients in group 1 received spironolactone versus 3 out of 18 (17%) patients in group 2, *p* = 0.062. There were no differences between the groups regarding the medication with acetylsalicylic acid, phenprocoumon, loop diuretics, ß-blockers, ACE inhibitors and amiodarone. Enteral budesonide capsules received 2 patients of group 1. Data regarding the history of PLE, chylothorax and plastic bronchitis (PB) are listed in Table 2.

**Table 2:** Frequency distribution of symptoms of PLE (protein-losing enteropathy), history of chylothorax, plastic bronchitis (PB) within the two groups.

	Group 1(*n* = 15) median (Q1;Q3)	Group 2 (*n* = 18) median (Q1;Q3)	
Echocardiographic parameters:			
AVVI	1 (0;1); *n* = 15	0.5 (0;1); *n* = 18	*p* = 0.485
AI	0 (0;0,1); *n* = 15	0 (0;0.25); *n* = 18	*p* = 0.770
MRI:			
EF	46 (40;54); *n* = 15	49.5 (44.3;56.0); *n* = 18	*p* = 0.356
EDVnorm (ml/m^2^)	96 (88;113); *n* = 15	94 (70.8;107.5); *n* = 18	*p* = 0.395
Treadmill CPET:			
Level	7.0 (6.0;8.0); *n* = 15	7(6:8); *n* = 18	*p* = 0.696
Distance (m)	720 (520;839); *n* = 15	646 (504;803.8); *n* = 18	*p* = 0.575
VO_2_max (ml/kg/min)	25.2 (22.6;29.7); *n* = 14	23.0 (20.4;26.4); *n* = 17	*p* = 0.234
O_2_ pulse (ml)	8.3 (6.2;11.4); *n* = 14	9.5 (8.0;14.1); *n* = 17	*p* = 0.266
Minimal oxygen saturation SaO_2_ during exercise	91 (86;93)	90 (86;93)	*p* = 0.921
RPA/LPA-Index:			
RPA mm^2^	173 (117;244)	197 (131;229)	*p* = 0.828
RPA/BSA	99 (72;137)	126 (113;138)	*p* = 0.120
LPA mm^2^	184 (132;240) *n* = 14	167 (102;251)	*p* = 0.676
LPA/BSA	107 (81;121) *n* = 14	96 (80;146)	*p* = 0.939

PLE occurred in 4 patients 9–10 years prior to the MRI-scan and in 1 patient 13 months prior to the MRI. This patient received TCPC at the age of 15,9 years. BP occurred in 2 patients 8–9 years prior to the MRI and in 2 patients about 2 years prior to the MRI scan. Results of liver ultrasound findings regarding liver structure, surface nodularity, splenomegaly, liver fibrosis, portal hypertension.

The liver parenchyma showed no significant differences in parenchymal structure regarding homogeneity of parenchyma and surface nodularity between both groups. There was also no significant difference in the occurrence of hepatomegaly and splenomegaly. In addition, both groups showed similar findings in echocardiographic parameters regarding atrioventricular valve insufficiency (AVVI) and aortic valve insufficiency (AI) (Table 3). Cardiac MRI parameters including ejection fraction (EF) and normalized end-diastolic volume (EDV) also showed no significant differences (Table 3). Data on exercise capacity are listed in Table 3 and showed no significant differences.

**Table 3:** Echocardiographic, MRI and treadmill CPET variables.

	Group 1(*n* = 15) median (Q1;Q3)	Group 2 (*n* = 18) median (Q1;Q3)	
NT-proBNP U/l	93 (52;281)	117 (90;232)	*p* = 0.527
Leucocytes 1/µl	6450 (5450;9690)	6655 (5512;7400)	*p* = 0.971
Haemoglobin g/dl	15.3 (14.3;16.4)	16.0 (15.7;16.6)	*p* = 0.107
Haematocrit %	43.4 (41.2;47.3)	45.5 (44.1;47.1)	*p* = 0.212
Lymphocytes Tsd/µl	1.210 (0.760;1.440)	1.425 (0.968;1.893)	*p* = 0.262
Platelets 1000/µl	196 (151;302)	199 (141;270)	*p* = 0.613
Platelet to lymphocyte ratio %	163 (141;250)	134 (106;168)	*p* = 0.076
Albumin g/dl	4.5 (3.7;4.5)	4.6 (0.4;4.6)	*p* = 0.117
Total bilirubin mg/dl	0.90 (0.7;1.7)	0.85 (1.04;0.68)	*p* = 0.744
AST U/l	27 (19;33)	22 (15;28)	*p* = 0.096
ALT U/l	38 (25;48)	32 (26;35)	*p* = 0.124
yGT U/l	63 (45;129)	68 (33;92)	*p* = 0.406
Calprotectin in stool µg/g	33 (24;93)	25 (24;88), *n* = 16	*p* = 0.411
α1-antitrypsin in stool mg/g	0.12 (0.06;0.17) *n* = 14	0.11(0.68;0.15) *n* = 14	*p* = 0.982

Minimal oxygen saturation during exercise (SaO_2_). Morphological measurement of RPA (right pulmonary artery area); LPA (left pulmonary artery area). RPA/BSA, LPA/BSA.

*EF* ejection fraction, *AVVI* atrioventricular valve insufficiency (0: none, 1: mild, 2: moderate, 3: severe), *AI* aortic valve insufficiency (0: none, 1: mild, 2: moderate, 3: severe), *EDVnorm* normalized end-diastolic volume, *VO*_*2*_*max* peak oxygen consumption.

**Fig. 4:**
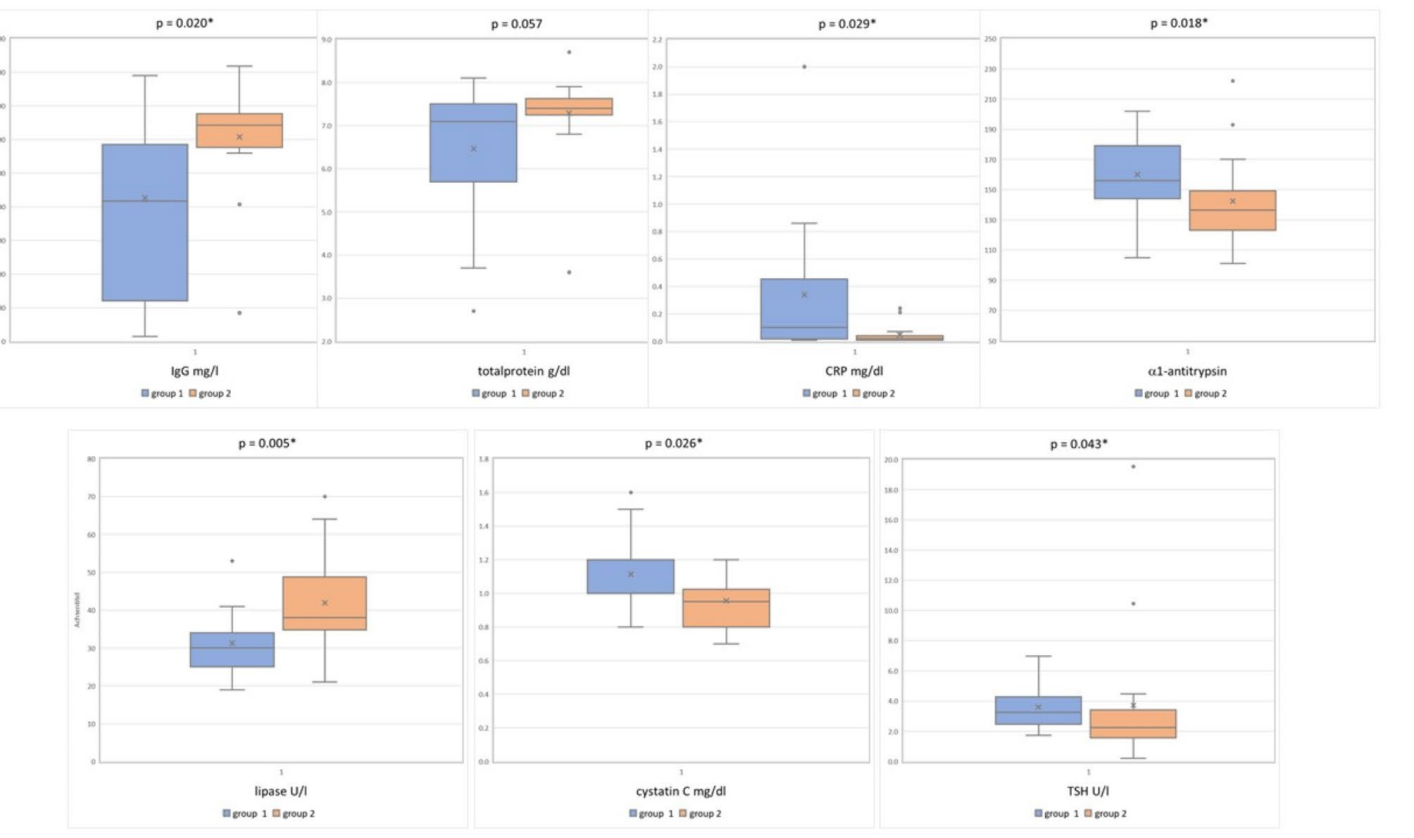
Comparison of blood sample values between both groups: IgG 833 (241;1170) mg/dl in group 1 vs. 1285 (1153;1354) mg/dl in group 2; total protein 7.1 (5.7;7.5) g/dl in group 1 vs. 7.4 (7.25;7.63) g/dl in group 2; CRP 0.1 (0.02;0.46) mg/dl in group 1 vs. 0.02 (0.01;0.04) mg/dl in group 2, α1-antitrypsin 156 (144;179) mg/dl in group 1 vs. 137 (123;149) mg/dl in group 2, lipase 30 (25;34) U/l in group 1 vs. 38 (35;49) U/l in group 2, Cystatin C 1.0 (1.0;1.2) mg/dl in group 1 vs. 0.95 (0.8;1.03) mg/dl in group 2, TSH 3.26 (2.46;4.28) U/l versus 2.26 (1.58;3.42) U/l in group 2.

Statistically significant results of the laboratory parameters and trends toward changes in total protein without statistically significance are depicted in Fig. 4. Laboratory parameters without any statistically significance are given in Table 4.

**Table 4:** Laboratory parameters.

	Group 1 (*n* = 15)	Group 2 (*n* = 18)	
PLE (history and current)	4	1	*p* = 0.092 Chi^2^(1) 2.836
Chylothorax (history)	7	2	p = 0.022* Chi^2^(1) 5.215
PB (history and current)	3	1	*p* = 0.206 Chi^2^(1) 1.603
Liver ultrasound findings:			
Heterogenous liver structure (y/n)	8/7	8/10	*p* = 0.611 Chi^2^(1) 0.259
Nodular surface (y/n)	7/8	8/10	*p* = 0.898 Chi^2^(1) 0.16
Splenomegaly (y/n)	7/6	6/9	*p* = 0.464 Chi^2^(1) 0.537
Liver fibrosis (y/n)	7/8	6/12	*p* = 0.435 Chi^2^(1) 0.609
Portal hypertension (y/n)	6/7	4/11	*p* = 0.283 Chi^2^(1) 1.152

Significant values are in bold.

All laboratory parameters collected are summarized in Supplemental Table 1. Comparison of alterations of the abdominal lymphatic vessels with changes of the cervical lymphatic vessels revealed that 5/15 (33.3%) patients in group 1 versus 3/18 (16.7%) patients in group 2 had cervical type 4 lymphatic alterations according to the score of Biko et al. (*p* = 0.266, Chi ^2^(1) = 1.237)^19^. There were no statistically significant differences in the absolute and on BSA related cross-sectional areas of the right and left pulmonary artery between the groups.

## Discussion

The number of patients with UVH following surgical establishment of a so-called Fontan circulation is constantly increasing^1–3,23^. In this patient population, late sequelae including protein losing enteropathy (PLE), plastic bronchitis, Fontan associated liver disease and alterations of the lymphatic system have an impact on life expectancy and quality of life^8,13,24–26^. In our study, we examined the potential pathophysiological role of alterations in the abdominal lymphatic vessels through an innovative imaging approach. Firstly, we developed a classification related to the morphology of abdominal lymphatic vessels. Secondly, we assessed the potential pathophysiological role of alteration in abdominal lymphatic vessels and clinical outcome parameters. Most importantly, we found a significant correlation between pronounced changes of the abdominal lymphatic system and a history of chylothorax and trends towards a more frequent history of PLE and plastic bronchitis. According to this finding, non-invasive lymphangiography might be suitable for early detection of a failing Fontan circulation.

Significant improvement in the noninvasive imaging of the thoracic and abdominal lymphatic system has been achieved by the introduction of heavily T2-weighted 3D FSE-sequences^15–18^. Biko et al. published a classification on different morphologies of the cervical lymphatic vessels in patients with UVH^19^. Patients with severe lymphatic abnormalities in the neck and the thorax following SCPC showed worse outcomes after TCPC^19,27^. In a previous study we found that patients with cervical lymphatic abnormalities type 4 in the long-term follow-up after TCPC showed reduced exercise tolerance and increased markers for failing Fontan circulation including PLE^9^.

There are few data so far concerning noninvasive imaging of abdominal lymphatic vessels in these patients. Shiina et al. investigated abdominal lymphatic vessels of Fontan patients based on gadolinium contrast with T2-weighted imaging^28^. Schroeder et al. examined 71 patients 6 months following TCPC with an age of 3 years (IQR:1)^15^. Noninvasive imaging of thoracic and abdominal lymphatic vessels was performed using a 2D-BLADE sequence. Classification of abdominal lymphatic perfusion patterns was performed based on the morphology of lymphatic vessels in the paraaortic and portal venous area^10^. Higher grade abnormalities of para-aortic lymphatics and higher-grade abnormalities of portal-venous lymphatic perfusion patterns correlated with higher-grade thoracic lymphatic abnormalities and were associated with decreased serum levels of total protein^10^. In our study, we followed a different approach with a specific focus on the differentiation of individual abdominal lymphatic vessels. Since 2D-BLADE sequences do not allow clear delineation of different lymphatic vessels we opted for heavily T2-weighted 3D FSE imaging and following MIP-reconstructions of 30 mm, which had been tested in a previous study in healthy volunteers^13^. Due to the different imaging techniques the classification of Schroeder et al. was not applicable in the present study. We decided to classify the morphology of abdominal lymphatic vessels in 3 categories: Based on the experience in healthy volunteers visualization of < 3 abdominal lymphatic was classified as type 1 and normal. The presence of 4–6 vessels was categorized as type 2 and the visualization of > 6 vessels as type 3 morphology. The latter morphology was considered as pronounced or high-grade abnormalities.

Based on this classification higher grade abdominal lymphatic abnormalities were not significantly associated with high grade thoracic lymphatic abnormalities in our cohort, (Supplemental Table 2). Patients with high-grade abnormalities of the abdominal lymphatic vessels showed significantly lower values for IgG and a trend towards lower serum protein levels without reaching statistical significance. Serum levels of Alpha1-antitrypsin, an acute-phase protein increasing with inflammation, were higher among patients with pronounced abdominal lymphatic abnormalities. Significantly lower values for lipase, and higher values for TSH in this group of patients appeared to be without critical clinical relevance. There were no differences regarding lymphocytes, platelets, or non-specific inflammatory parameters in blood or stool^29^. Finally, both groups showed no differences in mean pulmonary artery pressures, echocardiographic and cardiac MRI parameters. This is in accordance with previous studies which did not detect strong relationships between echocardiographic/ cardiac MRI findings and abnormalities of the lymphatic system^11,12,19^. However, in the present study we found a significant correlation between pronounced changes of the abdominal lymphatic system and a history of chylothorax and trends towards a more frequent history of PLE and plastic bronchitis. According to this finding noninvasive MR lymphangiography might be a suitable technique for early detection of a failing Fontan circulation^17,25,33]^.

Until now little is known concerning the possible timing of changes in lymphatic vessel morphology following the establishment of a Fontan-circulation. Moosmann et al. recently evaluated a cohort of 33 patients, who had undergone noninvasive MRI-imaging of the thoracic and abdominal lymphatic system at intervals of 7.5 months (IQR 33) and 4.5 years (IQR 3.6) following TCPC. Thoracic lymphatic vessels were evaluated according to the score of Biko et al., abdominal lymphatic abnormalities in the para-aortic and portal-venous region based on the score of Schroeder et al.^10,11,19^. During this relatively short time interval they could not demonstrate significant changes of lymphatic morphology among their patients^11^.

The present study as well as previous work describe morphology of lymphatic vessels following TCPC at a single point of time^10,19,20^. In comparison to other studies our cohort is characterized by a significantly older median age (19.8 years) and a longer median follow-up of 14.3 years after creation of the TCPC^10,11,19,20^. The comparison of patients with different degrees of severity of changes of abdominal lymphatic vessels in our cohort revealed that more pronounced cases were significantly younger at the time of MRI and had SCPC performed at a younger age. These findings are similar to previous results in the same cohort regarding changes of thoracic lymphatic vessels^9^. According to their younger age at the time of our study patients with more pronounced lymphatic abnormalities represent a cohort that has been treated more recently. Therefore, the duration of the time interval following creation of the Fontan circulation does not appear to be the single determinant of morphologic changes of the abdominal lymphatic vessels. The younger age at the time of SCPC among these younger patients reflects the general trend to perform the SCPC at a younger age during the recent two decades. However, there is concern that non-pulsatile pulmonary artery flow has a negative impact on pulmonary artery growth^30–32^. This negative impact might be more pronounced if the SCPC is performed at an earlier age and might result in detrimental long-term effects on the Fontan circulation, which is dependent on a well developed pulmonary vascular bed and a very low pulmonary vascular resistance. More pronounced changes of abdominal or thoracic lymphatic vessels might be the result of a less favorable anatomy of the peripheral pulmonary vascular bed resulting in congestion of the lymphatic system. However, until now this assumption is hypothetical and should prompt further evaluation in the future. The more frequent history of chylothorax and the laboratory finding of lower IgG values among patients with more pronounced abnormalities in our cohort might be a hint in this direction.

Studies on larger numbers of patients will be required to clarify the timing and progress of changes in the lymphatic system following establishment of a Fontan circulation and to correlate the morphologies with the onset of clinical signs of failing Fontan circulation like PLE, ascites, pleural effusions or Fontan associated liver disease. Highly resolved 3D T2-weighted MRI appears to be a valuable noninvasive tool to document the onset of both, thoracic and abdominal lymphatic vascular anomalies in patients with Fontan circulation.

### Limitations

This is a prospective single-center study describing the results of follow-up examinations at a single point of time. Due to the rather long time period changes in surgical techniques and medical treatment might have had an impact on the results. The younger patients were treated preferentially with a more recent modification of the TCPC, the extracardiac conduit while older patients preferentially underwent the so called intraatrial lateral tunnel. To avoid selection bias all patients, who had routine cardiac MRI for evaluation of TCPC, were offered MRL during the study period. However, some patients were lost during the long-term follow-up. Finally, another limitation is the relatively small sample size.

### Conclusion

The heavily T2-weighted 3D FSE sequence is a noninvasive MRI technique which can be performed without contrast agent under free-breathing condition to visualize the abdominal lymphatic system in patients with functionally univentricular hearts following establishment of a Fontan circulation. Due to its noninvasive nature this method appears to be a valuable tool to allow serial examinations in patients with Fontan circulation to clarify the onset and progress of morphologic changes of the abdominal and thoracic lymphatic system. In our cohort of patients with long-term follow-up after TCPC, patients with more pronounced changes of the abdominal lymphatic vessels were younger at the time of the MRI examination, younger at SCPC surgery and showed more frequently a history of chylothorax and lower IgG values.

## Electronic supplementary material

Below is the link to the electronic supplementary material.

Supplementary Material 1

Supplementary Material 2

Supplementary Material 3

Supplementary Material 4

Supplementary Material 5

Supplementary Material 6

## Data Availability

The datasets used and/ or analyzed during the current study are available from the corresponding author upon reasonable request.

## Abbreviations

AI: Aortic valve insufficiency
AVVI: Atrioventricular valve insufficiency
CHD: Congenital heart disease
CPET: Cardiopulmonary exercise test
CVP: Central venous pressure
EDV: End-diastolic volume normalized
EF: Ejection fraction
FALD: Fontan-associated liver disease
FSE: Fast-Spin-Echo
IgG: Immunoglobulin G
PLE: Protein-losing enteropathy
ROI: Vessel area region of interest
SCPC: Superior cavopulmonary connection
TCPC: Total cavopulmonary connection
VO_2_max: Maximal oxygen consumption

## References

[1] Rychik, J. et al. Evaluation and management of the child and adult with Fontan circulation: a Scientific Statement from the American Heart Association. *Circulation* :CIR0000000000000696. (2019). 10.1161/CIR.0000000000000696 31256636

[2] Plappert, L., Edwards, S., Senatore, A. & De Martini, A. The Epidemiology of Persons Living with Fontan in 2020 and projections for 2030: development of an epidemiology model providing multinational estimates. *Adv. Ther.* **39** (2), 1004–1015 (2022). 34936056 10.1007/s12325-021-02002-3 PMC8866255

[3] Pundi, K. N. et al. 40-Year Follow-Up after the Fontan Operation: long-term outcomes of 1,052 patients. *J. Am. Coll. Cardiol.* **66** (15), 1700–1710 (2015). 26449141 10.1016/j.jacc.2015.07.065

[4] Fontan, F. & Baudet, E. Surgical repair of tricuspid atresia. *Thorax* **26** (3), 240–248 (1971). 5089489 10.1136/thx.26.3.240 PMC1019078

[5] Kreutzer, G., Galindez, E., Bono, H., De Palma, C. & Laura, J. P. An operation for the correction of tricuspid atresia. *J. Thorac. Cardiovasc. Surg.* **66** (4), 613–621 (1973). 4518787

[6] Itkin, M. et al. Protein-losing enteropathy in patients with congenital heart disease. *J. Am. Coll. Cardiol.* **69** (24), 2929–2937 (2017). 28619193 10.1016/j.jacc.2017.04.023

[7] Dori, Y. et al. MRI of lymphatic abnormalities after functional single-ventricle palliation surgery. *AJR Am. J. Roentgenol.* **203** (2), 426–431 (2014). 24848564 10.2214/AJR.13.11797

[8] Gewillig, M. & Brown, S. C. The Fontan circulation after 45 years: update in physiology. *Heart* **102** (14), 1081–1086 (2016). 27220691 10.1136/heartjnl-2015-307467 PMC4941188

[9] Hanser, A. et al. Thoracic lymphatic anomalies in patients with univentricular hearts: correlation of morphologic findings in isotropic T2-weighted MRI with the outcome after fontan palliation. *Front. Cardiovasc. Med.* **10**, 1145613 (2023). 37229222 10.3389/fcvm.2023.1145613 PMC10203211

[10] Schroeder, C. et al. A classification of abdominal lymphatic perfusion patterns after Fontan surgery. *Eur. J. Cardiothorac. Surg.* **62**(4), ezac103 (2022). 10.1093/ejcts/ezac103 35218360

[11] Moosmann, J., Schroeder, C., Rompel, O., Purbojo, A. & Dittrich, S. Serial T2-Weighted thoracic and abdominal lymphatic imaging in Fontan patients-New insights into Dynamics of Lymphatic Abnormalities after total cavopulmonary connection. *J. Cardiovasc. Dev. Dis.* **9**(5), 138 (2022). 10.3390/jcdd9050138 PMC9144783 35621849

[12] Vaikom House, A. et al. Quantification of lymphatic burden in patients with Fontan circulation by T2 MR lymphangiography and associations with adverse Fontan status. *Eur. Heart J. Cardiovasc. Imaging* **24**(2), 241–249 (2023). 10.1093/ehjci/jeac216 36327421

[13] Poh, C. L. & d’Udekem, Y. Life after surviving Fontan surgery: a Meta-analysis of the incidence and predictors of late death. *Heart Lung Circ.* **27** (5), 552–559 (2018). 29402692 10.1016/j.hlc.2017.11.007

[14] Dunnick, N. R., Parker, B. R. & Castellino, R. A. Pediatric lymphography: performance, interpretation, and accuracy in 193 consecutive children. *AJR Am. J. Roentgenol.* **129** (4), 639–645 (1977). 409237 10.2214/ajr.129.4.639

[15] Dori, Y. Novel lymphatic imaging techniques. *Tech. Vasc Interv Radiol.* **19** (4), 255–261 (2016). 27993320 10.1053/j.tvir.2016.10.002

[16] Takahashi, H. et al. Clinical feasibility of noncontrast-enhanced magnetic resonance lymphography of the thoracic duct. *Chest* **124** (6), 2136–2142 (2003). 14665492 10.1378/chest.124.6.2136

[17] Arrive, L. et al. Noncontrast Magnetic Resonance Lymphography. *J. Reconstr. Microsurg*. **32** (1), 80–86 (2016). 25826439 10.1055/s-0035-1549133

[18] Hanser, A. et al. T2-Weighted High-Resolution Isotropic magnetic resonance lymphangiography of the thoracic and abdominal lymphatic vessels with and without previous high-Fat Meal. *Acad. Radiol.* **28** (Suppl 1), S218–S224 (2021). 33183951 10.1016/j.acra.2020.10.008

[19] Biko, D. M. et al. MRI Evaluation of Lymphatic Abnormalities in the Neck and Thorax after Fontan surgery: relationship with outcome. *Radiology* **291** (3), 774–780 (2019). 30938628 10.1148/radiol.2019180877 PMC6542623

[20] Dittrich, S. et al. Association of Lymphatic Abnormalities with early complications after Fontan Operation. *Thorac. Cardiovasc. Surg.* **69** (S 03), e1–e9 (2021). 33383591 10.1055/s-0040-1722178 PMC7909602

[21] Dubowy, K. O., Baden, W., Bernitzki, S. & Peters, B. A practical and transferable new protocol for treadmill testing of children and adults. *Cardiol. Young*. **18** (6), 615–623 (2008). 18838025 10.1017/S1047951108003181

[22] Riordan, R. D., Khonsari, M., Jeffries, J., Maskell, G. F. & Cook, P. G. Pineapple juice as a negative oral contrast agent in magnetic resonance cholangiopancreatography: a preliminary evaluation. *Br. J. Radiol.* **77** (924), 991–999 (2004). 15569640 10.1259/bjr/36674326

[23] Dittrich, S. et al. German Registry for Cardiac Operations and interventions in patients with congenital heart disease: Report 2021 and 9 years’ longitudinal observations on Fallot and Coarctation patients. *Thorac. Cardiovasc. Surg.* **70** (S 03), e21–e33 (2022). 36174655 10.1055/s-0042-1757175 PMC9536750

[24] Kaulitz, R., Haber, P., Sturm, E., Schafer, J. & Hofbeck, M. Serial evaluation of hepatic function profile after Fontan operation. *Herz* **39** (1), 98–104 (2014). 23649317 10.1007/s00059-013-3811-5

[25] Brown, M. J. et al. Imaging of Fontan-Associated Liver Disease. *J. Comput. Assist. Tomogr* **48**(1), 1–11 (2023) 10.1097/RCT.0000000000001533 37574655

[26] de Lange, C., Moller, T. & Hebelka, H. Fontan-associated liver disease: diagnosis, surveillance, and management. *Front. Pediatr.* **11**, 1100514 (2023). 36937979 10.3389/fped.2023.1100514 PMC10020358

[27] Ghosh, R. M. et al. Prevalence and cause of early Fontan complications: does the lymphatic circulation play a role? *J. Am. Heart Assoc.* **9** (7), e015318 (2020). 32223393 10.1161/JAHA.119.015318 PMC7428641

[28] Shiina, Y. et al. Abdominal lymphatic pathway in Fontan circulation using non-invasive magnetic resonance lymphangiography. *Heart Vessels*. **38** (4), 581–587 (2023). 36318300 10.1007/s00380-022-02196-8

[29] Al Balushi, A. & Mackie, A. S. Protein-losing Enteropathy following Fontan Palliation. *Can. J. Cardiol.* **35** (12), 1857–1860 (2019). 31711823 10.1016/j.cjca.2019.07.625

[30] Ovroutski, S. et al. Absence of pulmonary artery growth after fontan operation and its possible impact on late outcome. *Ann. Thorac. Surg.* **87** (3), 826–831 (2009). 19231398 10.1016/j.athoracsur.2008.10.075

[31] Buheitel, G. et al. Changes in pulmonary artery size before and after total cavopulmonary connection. *Heart* **78** (5), 488–492 (1997). 9415009 10.1136/hrt.78.5.488 PMC1892292

[32] Unseld, B. et al. An early Glenn Operation May be Associated with the later occurrence of protein-losing Enteropathy in Fontan patients: Association of Early Glenn and Failing Fontan. *Pediatr. Cardiol.* **38** (6), 1155–1161 (2017). 28534240 10.1007/s00246-017-1632-7

