# Supplementary material for: Feasibility, classification and potential clinical impact of non-invasive delineation of abdominal lymphatic vessels in patients following TCPC with T2 weighted MRI
Source: Sci Rep. 2024 Nov 29;14:29752. doi: 10.1038/s41598-024-81299-w (PMC11607435; doi:10.1038/s41598-024-81299-w)
Supplement: Supplementary file 1 — Supplementary Material 1 [file 41598_2024_81299_MOESM1_ESM.docx]

|  | **Abdominal Lymphatic Vessels** | |  | **Cervical Lymphatic Vessels** | |  |
| --- | --- | --- | --- | --- | --- | --- |
|  | **group 1 (n=15)**  **type 3**  **median (Q1;Q3)** | **group 2 (n=18)**  **type 1+2**  **median (Q1;Q3)** |  | **group 1 (n=8)**  **type 4**  **median (Q1;Q3)** | **group 2 (n=25)**  **type 1-3**  **median (Q1;Q3)** |  |
| **Age SCPC/Glenn (m)** | **9.9** (8.0;25.6) | **29.0** (13.7;66.6) | **p = 0.018*** | **11.3** (8.3;21.5) | **25.6** (9.8;63.3) | **p = 0.083** |
| **Age at TCPC (y)**  **one stage Fontan** | **3.8** (3.2;4.7)  n = 4 | **4.2** (2.5;6.7)  n = 7 | p = 0.691 | **3.1** (1.7;12.9)  n = 1 | **4.2** (3.2;6,2)  n = 10 | p = 0.220 |
| **SPCP-TCPC (m)** | **30.6** (0;41.9) | **12.2** (0;25.4) | p = 0.131 | **26.1** (2.9;145.0) | **15.2** (0;33.7) | p = 0.245 |
| **Age at MRI (y)** | **17.4** (14.3;18.9) | **26.2** (18.2;32.3) | **p = 0.030*** | **16.8** (12.3;24.8) | **20.6** (16.3;31.1) | p = 0.074 |
| **Follow-up time TCPC-MRI (y)** | **12.9** (9.6;16.1) | **23.2** (9.7;26.2) | p = 0.083 | **10.4** (4.7;20.3) | **17.3** (12.3;25.5) | **p = 0.044*** |
