# Supplementary material for: Feasibility, classification and potential clinical impact of non-invasive delineation of abdominal lymphatic vessels in patients following TCPC with T2 weighted MRI
Source: Sci Rep. 2024 Nov 29;14:29752. doi: 10.1038/s41598-024-81299-w (PMC11607435; doi:10.1038/s41598-024-81299-w)
Supplement: Supplementary file 6 — Supplementary Material 6 [file 41598_2024_81299_MOESM6_ESM.docx]

**Supplementary Legends:**

**Electronic Supplementary Material:**

**Supplement Table 1: Overview of all laboratory parameters collected**

**Supplement Table 2: Data regarding age of patients at surgical steps and follow-up time in patients with abdominal lymphatic abnormalities and cervical lymphatic abnormalities** [9, 19]. SCPC= superior cavopulmonary connection, TCPC = total cavopulmonary connection.

**Movie 1: patient 11:** 30 years, male, hypoplastic left heart, lateral tunnel, asymptomatic

**Movie 2: patient 19:** 39 years, female, DILV (double inlet left ventricle), atriopulmonary connection, reduced exercise capacity

**Movie 3: patient 25:** 17 years, male, tricuspid atresia, extracardiac conduit, protein-losing Enteropathy, AV block III
